# Pancancer Analyses of KISS1 as a Potential Biomarker for Tumor Metastasis and Immunotherapy and Therapeutic Target for Breast Cancer

**DOI:** 10.1155/ijog/5902518

**Published:** 2026-01-08

**Authors:** Chunbiao Wu, Hao Jiang, Wei Xu, Bo Li, Hao Zhang, Long Zhang, Zhenxi Li, Jianru Xiao

**Affiliations:** ^1^ Institute of Orthopedic Biomedical and Device Innovation, School of Health Science and Engineering, University of Shanghai for Science and Technology, Shanghai, China, usst.edu.cn; ^2^ Department of Orthopedic Oncology, Shanghai Changzheng Hospital, Naval Medical University, Shanghai, China, smmu.edu.cn

**Keywords:** biomarker, breast cancer, immune infiltration, KISS1, posttranslational modification, tumor metastasis

## Abstract

Emerging evidence highlights the pivotal role of KISS1 in cancer metastasis; however, there remains a dearth of pancancer analyses, particularly concerning immunotherapy. Here, we conducted a comprehensive investigation of KISS1 across various cancers, with a specific focus on breast cancer, using TCGA and GTEx datasets. We observed a tissue context‐dependent role function of KISS1 in tumor metastasis, which exhibited suppressive effects in various tumors but promoted the metastatic phenotype in breast cancer. Our study revealed a noteworthy disparity between KISS1 expression at the mRNA and protein levels, indicating potential posttranslational modifications within cancer cells. Moreover, KISS1 is significantly associated with immune cell infiltration and immunosuppressive cells, suggesting its crucial role in modulating tumor immunotherapy. Intriguingly, our investigation also elucidated KISS1’s involvement in promoting breast cancer metastasis, thereby providing valuable insights into the molecular underpinnings of this process. Furthermore, we validated the presence of posttranslational modifications of KISS1 in breast cancer, adding to our understanding of its role in tumorigenesis. By shedding light on the tissue context‐dependent function of KISS1 and its implications for immunotherapy, our pancancer study offers novel perspectives on the oncogenic roles of KISS1 and provides potential avenues for the development of targeted therapies and diagnostic biomarkers.

## 1. Introduction

The durable control of cancers remains a challenge for medical workers, especially when metastases develop, although there has been a revolution in cancer treatment and early detection [1]. Metastasis to other organs, even a preference for some specific organs [2], represents a major obstacle in cancer therapies [3]. Drug resistance of tumor cells accounts for the major reason that leads to an unsatisfactory outcome for neoplastic disease treatment [4], and drug resistance is associated with the promotion of metastatic ability with the occurrence of epithelial‐to‐mesenchymal transition (EMT) [5]. The development and outgrowth of cancer metastasis depend on several positive and negative regulators [6], including metastasis suppressor genes or proteins that are subjected to modifications during gene transcription and protein translation [7]. Thus, several metastasis suppressors have been described [8], and among them, KISS1 raised our interest for its still controversial role in tumor metastasis [9].

Physiologically, KISS1 binds to the G protein‐coupled receptor (GPR54, also known as KISS1R) to activate the hypothalamic–pituitary–gonadal axis, which regulates puberty and reproductive functions [10]. Originally, its metastasis suppressor role was identified in melanoma cells, in which cancer metastasis was suppressed by the introduction of the human chromosome 6 [11]. Concordantly, other studies have confirmed the metastasis–suppressive role of KISS1 in the lung, prostate, and pancreas, mainly due to the KISS1 receptor and the negative regulation of CXCR4 (CXC chemokine receptor 4) [12]. However, KISS1 has been reported to promote the metastatic capacity of estrogen receptor (ER) negative mammary epithelial and breast cancer cells [13]. Therefore, the role in cancer metastasis of KISS1 is context‐dependent, and further studies are needed to elucidate the underlying mechanisms.

Given all the previous studies, there are no studies on KISS1 in the entire cancer spectrum. Here, we conducted a pancancer study using public databases to acquire a more efficient understanding of KISS1 in terms of its molecular mechanisms and predictive value in tumor biology [14]. Technologically, we included a number of factors, such as gene expression, protein expression, survival status, immune infiltration, immunosuppressive cells, cell experiments, and posttranslational modifications (PTMs) to investigate the potential molecular mechanism of KISS1 in the pathogenesis and clinical prognosis of various cancers [14].

## 2. Materials and Methods

### 2.1. mRNA and Protein Expression Analysis

For mRNA expression, we downloaded 10,535 pancancer samples from The Cancer Genome Atlas (TCGA) and 15,776 data combined with the Genotype‐Tissue Expression (GTEx) (https://commonfund.nih.gov/GTEx/) from the University of California, Santa Cruz (UCSC) (https://xenabrowser.net/) database. The mRNA sequencing data for KISS1 in 34 tumors and 31 normal tissues were transformed by log2(*x* + 0.001) [15]. The protein expression analysis of the CPTAC (Clinical Proteomic Tumor Analysis Consortium) dataset was conducted on the UALCAN portal (the University of ALabama at Birmingham CANcer data analysis Portal) (http://ualcan.path.uab.edu/analysis-prot.html) [16]. In addition, we explored protein expression status using immunohistochemical samples from the Human Protein Atlas (HPA) web (https://www.proteinatlas.org/) [17].

### 2.2. Correlations Between KISS1 Expression and Clinical Features

Analysis of the relationship between clinical stage, metastasis status, and KISS1 expression was performed using R software (Version3.6.4) and obtained violin plots of KISS1 expression in different pathological stages of some indicated tumors using the GEPIA2 (Gene Expression Profiling Interactive Analysis, Version 2) web server (http://gepia2.cancer-pku.cn/#analysis) [18].

### 2.3. Survival Prognosis Assessment

The prognostic value of KISS1 in various cancers was assessed by two types of clinical outcomes: overall survival (OS) and disease‐free survival (DFS). High‐quality prognostic expression data were picked up from the pancancer expression data, and the results were visualized using “Quick select” section on the cBioPortal web (https://www.cbioportal.org/) [19].

### 2.4. Relationship Analysis of Immune Traits

We explored the association between KISS1 and immune infiltrates in pancancer from TCGA by the “Immune‐Gene” module of the TIMER2 (Tumor Immune Estimation Resource) web server (http://timer.cistrome.org/) [20]. The StromalScore, ImmuneScore, and ESTIMATE scores were obtained using the R package “ESTIMATE” for all tumor samples [21]. KISS1 and 8 immune checkpoint pathway genes were collected from a pancancer gene expression dataset [22]. Regarding the relationship with immunosuppressive cells, we selected myeloid‐derived suppressor cells (MDSCs) and regulatory T cells (Tregs) for analysis [22]. The CIBERSORT, CIBERSORT‐ABS, XCELL, and EPIC algorithms were applied for immune infiltration estimations [14]. We performed the purity‐adjusted Spearman’s rank correlation test to obtain the *p* values and partial correlation values. These data are demonstrated as a heatmap and scatter plot.

### 2.5. Cell Culture, Transfections, Immunoblotting (IB), and Immunoprecipitation

MDA‐MB‐231 was purchased from the American Type Culture Collection (ATCC), and highly bone‐metastatic (BM) derivatives were obtained from Dr. Zhenxi LI (Changzheng Hospital, 415 Feng Yang Road, Shanghai 200003, People’s Republic of China). All cell lines were authenticated by STR profiling and compared with results in the ATCC database. Also, the cell lines were tested for mycoplasma contamination using GENEWIZ. The cells were cultured in DMEM (BasalMedia, L110KJ) supplemented with 10% FBS (Ylesa, S211201) and 1% 100x penicillin–streptomycin solution (Yeasen, 60162ES76) and grown at 37°C in a 5% CO_2_ incubator. Human KISS1 was cloned into the pLVX‐neo vector (Clontech) for overexpression, and annealed sense and antisense shRNA oligonucleotides were cloned into the pLKO.1‐puro vector (Addgene) for the knockdown of human KISS1. The shRNAs used were TRCN0000059065 (#1), TRCN0000059064 (#2), and TRCN0000059067 (#3). Flag‐WWP1 was transfected into MDA‐MB‐231 cells using PEI (Polysciences, 24765‐100) according to the manufacturer’s protocol. During immunoprecipitation assays, cells were lysed with RIPA (Beyotime, P0013D) containing protease inhibitors (Sigma), and immunoprecipitations were performed using anti‐Flag M2 beads (Sigma, M8823) at 4°C. The immunoprecipitates were washed with NETN buffer. IB analysis was performed using specific antibodies and related secondary antibodies, and visualization was obtained using chemiluminescence. The antibodies used in IB analysis were anti‐KISS1 (Sigma‐Aldrich, MABC60), anti‐GAPDH (Abcam, ab8245), and anti‐Flag (Abcam, ab205606).

### 2.6. Cell Colony Formation and Transwell Assay

Cells were positioned on a six‐well plate and cultured for 2 weeks, during which time the medium was changed every 3 days. Formed colonies were fixed in 4% paraformaldehyde for 30 min, stained with crystalline violet (Sigma‐Aldrich, C0775) for 10 min, and photographed and analyzed by ImageJ software. As for the transwell assay, 8000 cells were inoculated into each transwell chamber (Corning, CLS3378), with 200 *μ*L DMEM containing 1% FBS, and 600 *μ*L culture medium containing 20% FBS was added to the lower chamber.

### 2.7. Nude Mice Experiments

All animal experiments were conducted in strict accordance with the ethical guidelines of the Experimental Animal Ethics Committee of Yangzhou University (Approval No. 202504008) and followed the ARRIVE guidelines for reporting animal research. Six‐week‐old female BALB/c nude mice were housed under specific pathogen‐free (SPF) conditions with a 12‐hour light/dark cycle and provided ad libitum access to food and water. Mice were anesthetized with isoflurane (2‐3% induction, 1‐1.5% maintenance in oxygen) to minimize pain and distress during procedures. Euthanasia was performed via gradual carbon dioxide inhalation in compliance with the guidelines of the American Veterinary Medical Association (AVMA) for humane euthanasia: medical‐grade CO_2_ was delivered at a flow rate of 10–30% of the chamber volume per minute to gradually increase the concentration to 70–80% (avoiding rapid CO_2_ elevation, which may cause respiratory distress). The mice were maintained in the CO_2_ atmosphere for at least 5 minutes after the cessation of spontaneous respiration. Death was further confirmed by the absence of heartbeat (via palpation of the chest) and pupillary reflex to ensure no residual vital signs. Intra‐iliac artery injections were performed as previously described [23]. In brief, 1 × 10^6^ luciferase‐labeled cells were injected in into right iliac artery.

### 2.8. Statistical Analysis

Gene expression differences were compared using the Kruskal–Wallis and Wilcoxon rank‐sum tests. Correlations between the two groups were calculated using the Spearman or Pearson correlation analysis. Survival curves were generated using the Kaplan–Meier (KM) method and Cox regression analysis. Categorical variables were analyzed using the chi‐square test and Fisher’s exact test. Statistical significance was determined using the log‐rank test, and the level of significance was set at  ^∗^ *p* < 0.05,  ^∗∗^ *p* < 0.01, and  ^∗∗∗^ *p* < 0.001. The above visualization was performed using R software (Version 3.6.4), GraphPad Prism 9, and Sangerbox web (http://www.sangerbox.com/) [24].

## 3. Results

### 3.1. Gene Expression of KISS1 in Pancancer

To obtain the expression status of KISS1 in pancancer, we analyzed 33 cancers based on TCGA database. The KISS1 was significantly upregulated in 13 cancers, including LUAD, COAD, COADREAD, BRCA, STES, STAD, UCEC, LIHC, THCA, READ, PAAD, BLCA, and CHOL (Figure 1a) (abbreviations could be seen in Supporting Information 1: File S1). However, we also found that the KISS1 was downregulated in KIRP, KIPAN, and LUSC (Figure 1a). Furthermore, we explored the GTEx database to evaluate the difference in KISS1 expression between normal and tumor tissues. As shown in Figure 1b, the KISS1 was upregulated in 19 cancers, such as UCEC, BRCA, CESC, LUAD, ESCA, STES, COAD, COADREAD, STAD, LIHC, BLCA, THCA, READ, OV, PAAD, UCS, ALL, ACC, and CHOL, and downregulated in 13 cancers, including GBM, GBMLGG, LGG, KIRP, KIPAN, PRAD, KIRC, LUSC, WT, SKCM, TGCT, LAML, and KICH (Figure 1b).

Figure 1Expression level of KISS1 mRNA and protein in pancancer. (a) The expression status of KISS1 gene in tumor tissues by data from TCGA database. (b) Analysis of KISS1 gene expression between tumor tissues from TCGA and corresponding normal tissues from GTEx database. Ns, *p* ≥ 0.05;  ^∗^ *p* < 0.05;  ^∗∗^ *p* < 0.01;  ^∗∗∗^ *p* < 0.001;  ^∗∗∗∗^ *p* < 0.0001. (c) The KISS1 expression of human normal tissues in immunohistochemical from HPA dataset. (d) The protein expression of KISS1 in human cancer tissues from HPA dataset.

**Figure:**
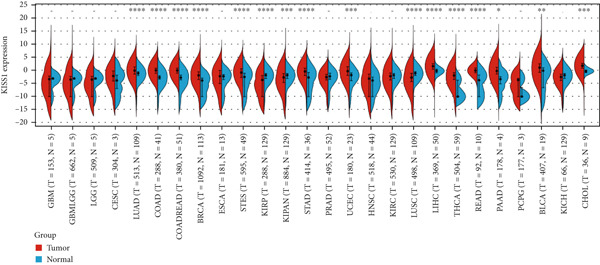
(a)

**Figure:**
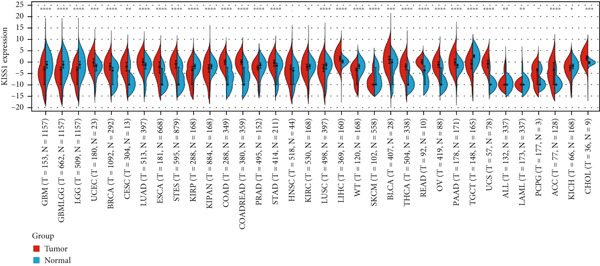
(b)

**Figure:**
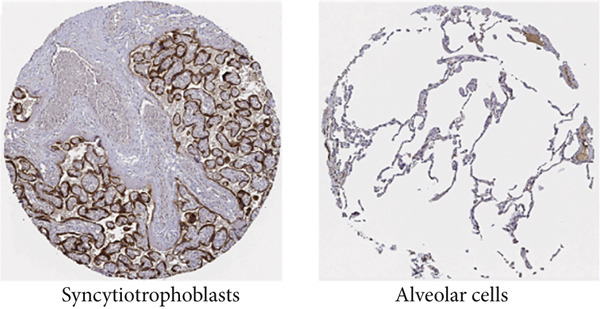
(c)

**Figure:**
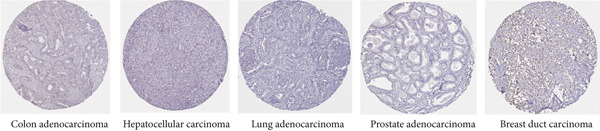
(d)

### 3.2. Protein Expression of KISS1 in Pancancer

These results made us interested in the protein expression status of KISS1 in various cancers. Unfortunately, there is no public database available from the CPTAC dataset [16], which indicates that the recognition of KISS1 remains superficial. In terms of the protein expression level in human tissues, KISS1 could be physiologically detected in tissues like syncytiotrophoblasts (HPA035542), mainly located in the cytoplasmic and membranous compartments within cells, but nearly no expression was observed in other tissues like lung (CAB017775) (Figure 1c). Surprisingly, according to the databases in the HPA database, except for breast duct carcinoma (CAB017775), no KISS1 protein was detected in cancers like colon adenocarcinoma (HPA035542), hepatocellular carcinoma (HPA035542), lung adenocarcinoma (HPA035542), and prostate adenocarcinoma (HPA035542) (Figure 1d). The contradictory results between mRNA and protein expression in pancancers call for further investigation of KISS1, especially in terms of PTMs.

### 3.3. Expression Analysis of KISS1 in Different Pathological and Clinical Stages

In a prospective view of pancancer, we assessed the importance of KISS1 expression at different clinical and pathological stages. In COAD, COADREAD, BRCA, KIRP, KIPAN, and KIRC, KISS1 demonstrated a differential expression status in different clinical and pathological stages (Figure 2a). Detailed information on COAD, COADREAD, BRCA, KIRP, KIPAN, and KIRC is shown in Figure 2b. In addition, as KISS1 has been reported as a metastasis suppressor [8], we investigated the expression differences between different metastasis statuses in pancancers. Interestingly, according to datasets from TCGA, only in COADREAD, KIPAN, HNSC, PAAD, and SKCM does it make sense (Figure 2c); the detailed information is shown in Figure 2d. However, KISS1 has been reported to suppress the metastasis of various cancers, such as melanoma, bladder cancer, and lung cancer [10]. These contradictions indicate that the analysis of KISS1 is underexplored, especially in terms of its prognostic value.

Figure 2Expression analysis of KISS1 in different pathological and clinical stages. (a, b) KISS1 expression at different clinical stages. (c, d) KISS1 expression at different metastasis statuses.

**Figure:**
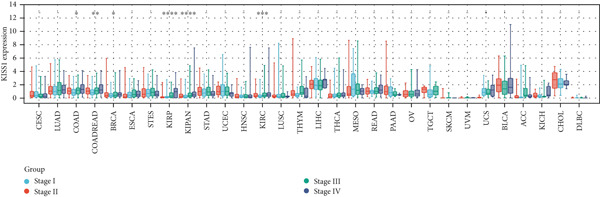
(a)

**Figure:**
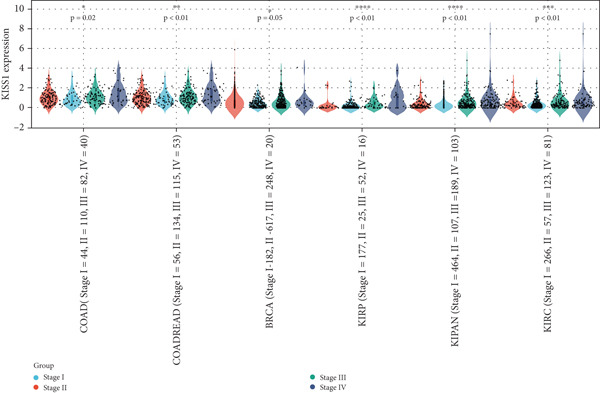
(b)

**Figure:**
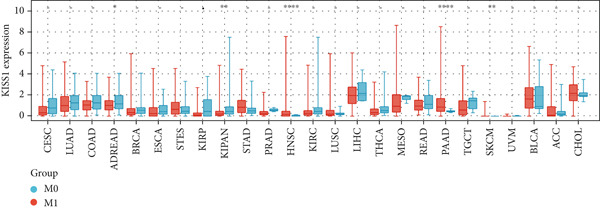
(c)

**Figure:**
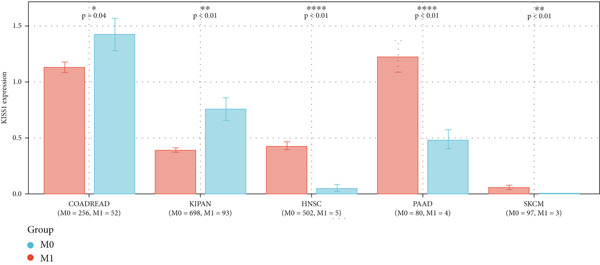
(d)

### 3.4. Survival Analysis

For prognostic value, we first adopted the Cox regression model and KM survival analysis of KISS1 in OS and DFS [14]. According to the expression levels of KISS1, all the cancer cases in TCGA and GEO were divided into two groups: high‐ and low‐expression groups. We then investigated the correlation between KISS1 expression and patient prognosis from these datasets. Within TCGA project, high expression of KISS1 contributed to a poor prognosis of OS for cancers of KIRP, KIRC, and LUAD. However, the poor prognosis of OS in BLCA patients is related to the low expression of KISS1 (Figure 3a). Besides, high KISS1 expression was a risk factor for DFS in patients with CESC, COAD, KIRP, and PAAD (Figure 3b). These relationships between KISS1 and OS and DFS were further demonstrated by KM survival analysis curves (Figure 3a,b). In comparison, KISS1 expression was correlated with both OS and DFS in KIRP.

Figure 3Relationship between KISS1 expression and survival prognosis of pancancers in TCGA. (a) Survival map and Kaplan–Meier curves of OS (overall survival) of various tumors by KISS1 gene expression. (b) Survival map and Kaplan–Meier curves of DFS (disease‐free survival) of various tumors by KISS1 gene expression. (c, d) Prognostic significance of KISS1 in breast cancer by univariate and multifactorial COX analyses.

**Figure:**
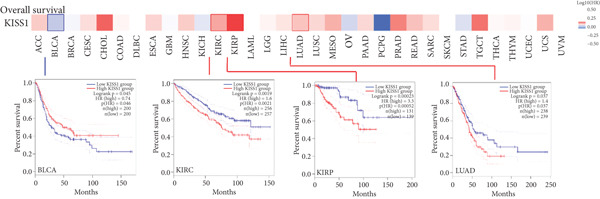
(a)

**Figure:**
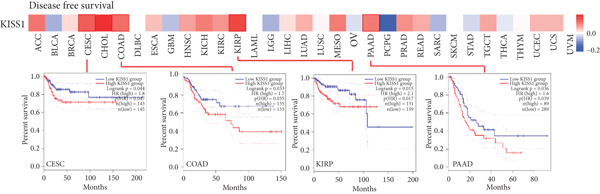
(b)

**Figure:**
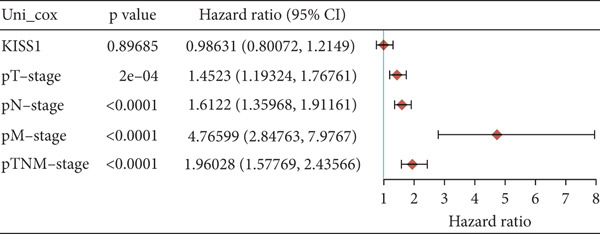
(c)

**Figure:**
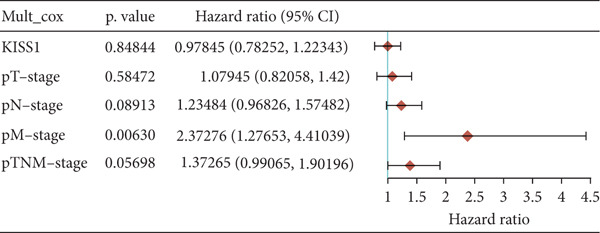
(d)

### 3.5. KISS1 Is Significantly Related to Immune Infiltration

To investigate the potential role of KISS1 in the tumor microenvironment (TME), we explored the relationship between KISS1 and the level of immune infiltration in various cancers from TCGA using three immune scores and selected the four most significant ones. First, according to the StromalScore [21], KISS1 expression in STAD (*N* = 388, *R* = −0.25, *p* = 6.8e − 7), BRCA (*N* = 1077, *R* = −0.13,*p* = 2.6e − 5), and LIHC (*N* = 363, *R* = −0.25, *p* = 1.5e − 6) was negatively correlated with immune infiltration (Figure 4a). In HNSC (*N* = 517, *R* = 0.15, *p* = 5.6e − 4), KISS1 expression positively correlated with immune infiltration (Figure 4a). Then, as for the ImmuneScore [21], the expression of KISS1 was significantly negatively correlated with immune infiltration in COAD (*N* = 282, *R* = −0.20, *p* = 6.1e − 4), COADREAD (*N* = 373, *R* = −0.21, *p* = 4.2e − 5), and STAD (*N* = 388, *R* = −0.30, *p* = 1.4e − 9) and positively correlated with immune infiltration in PRAD (*N* = 495, *R* = 0.20, *p* = 5.1e − 6) (Figure 4b). Lastly, by the EstimateScore, KISS1 expression in SARC (*N* = 258, *R* = −0.22, *p* = 4.1e − 4), STAD (*N* = 388, *R* = −0.30, *p* = 1.4e − 9), and LIHC (*N* = 363, *R* = −0.20, *p* = 1.4e − 4) was negatively correlated with immune infiltration (Figure 4c). There was a positive correlation between KISS1 expression and immune cell infiltration in PRAD (*N* = 495, *R* = 0.19, *p* = 2.6e − 5) (Figure 4c). In summary, we found a consistent trend that KISS1 negatively regulates TME in most cancers from TCGA, and KISS1 expression and immune infiltration were both highly expressed in cancers such as PRAD. However, the relationship between KISS1 expression and immune infiltration requires further investigation before we can make a clear conclusion.

Figure 4Relationship between KISS1 expression and immune infiltration. (a) Correlation of KISS1 expression with StromalScore. (b) Correlation of KISS1 expression with ImmuneScore. (c) Correlation of KISS1 expression with EstimateScore.

**Figure:**
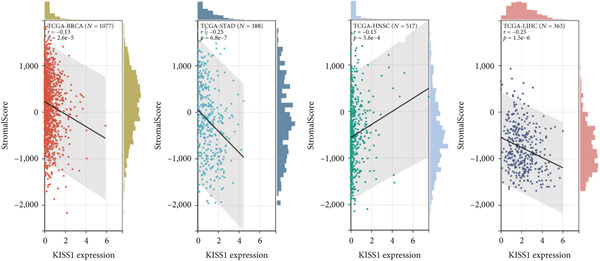
(a)

**Figure:**
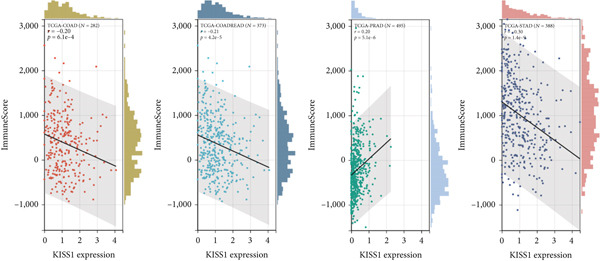
(b)

**Figure:**
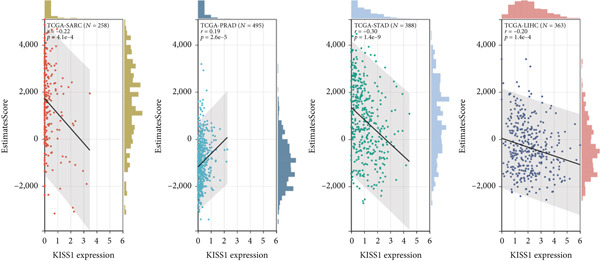
(c)

### 3.6. KISS1 Interacts Evidently With Immunosuppressive Cells

Previously, we found that KISS1 expression may negatively regulate TME. Furthermore, we explored the importance of KISS1 in the suppressive tumor microenvironment (STM) through the relationship between KISS1 expression and the infiltration level of MDSCs and Tregs [25]. As a result, we observed a statistically significant positive correlation of KISS1 expression and immune infiltration of MDSCs in tumors of BRCA, BRCA‐Basal, BRCA‐LumA, BRCA‐LumB, CESC, KIRC, KIRP, LGG, LIHC, MESO, PAAD, SARC, SKCM, SKCM‐Metastasis, STAD, TGCT, and UVM but also a negative correlation for tumors only in BLCA and ESCA (Figure 5a), based on TIED algorithms. The scatterplot data of BLCA, ESCA, BRCA, BRCA‐Basal, BRCA‐LumA, BRCA‐LumB, PAAD, and STAD are listed in Figure 5b.

Figure 5Correlation analysis between KISS1 expression and immunosuppressive cell infiltration. (a, b) Correlation between KISS1 expression and myeloid‐derived suppressor cell (MDSC) infiltration. (c, d) Correlation between KISS1 expression and regulatory T cell (Tregs) infiltration. (e) Relationship between KISS1 and chemokine genes.

**Figure:**
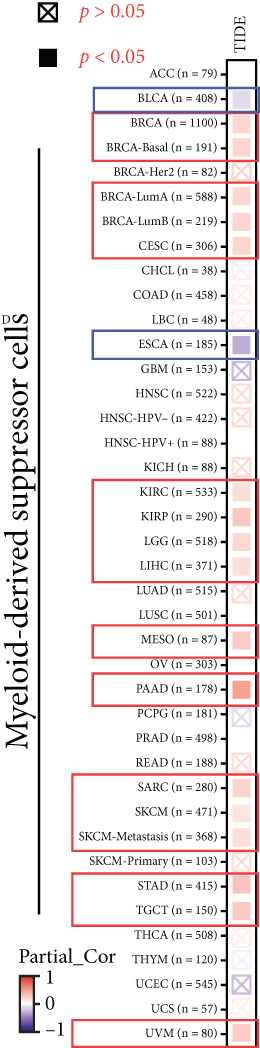
(a)

**Figure:**
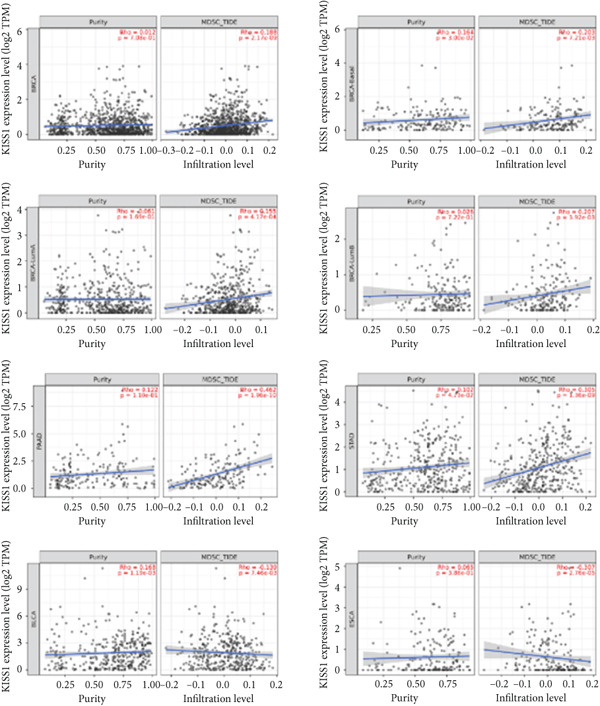
(b)

**Figure:**
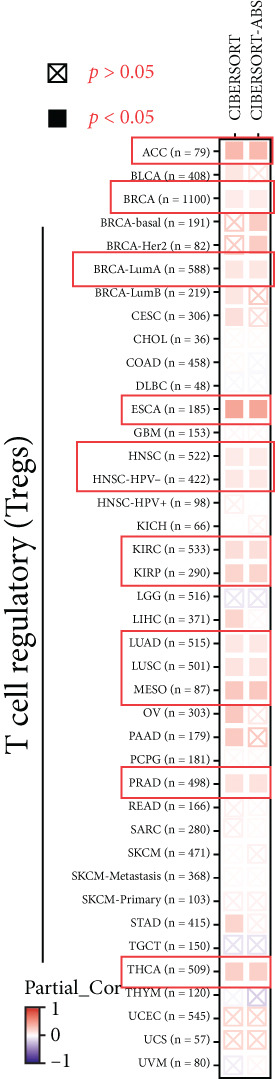
(c)

**Figure:**
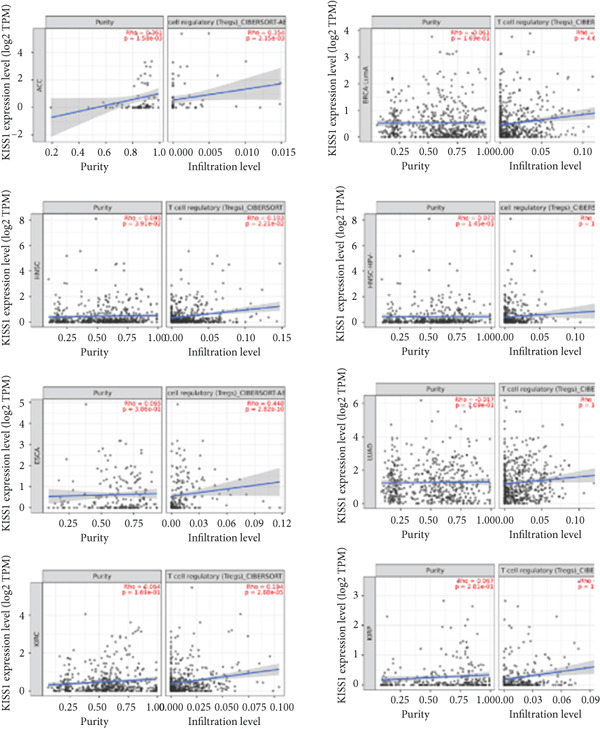
(d)

**Figure:**
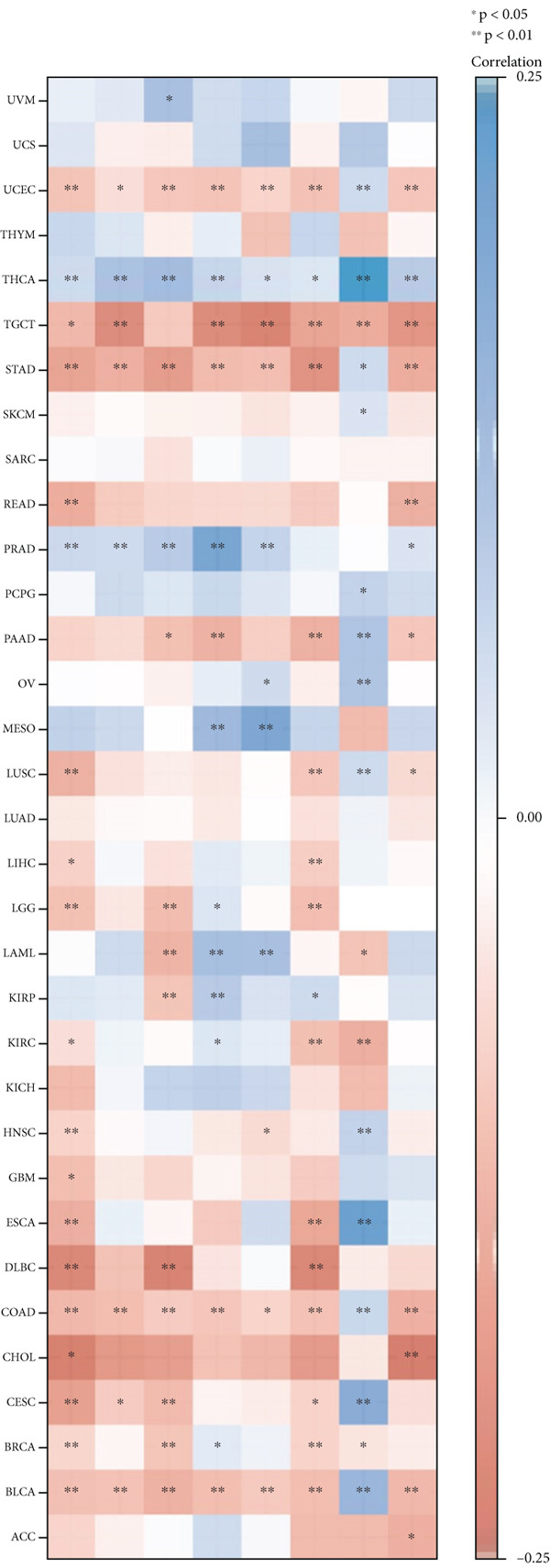
(e)

Following by MDSCs, we analyzed the relationship between KISS1 expression and Tregs infiltration. According to the CIBERSORT and CIBERSORT‐ABS algorithms, we found a statistically positive correlation between the immune infiltration of Tregs and KISS1 expression in tumors of ACC, BRCA, BRCA‐LumA, ESCA, HNSC, HNSC (HPV [human papillomavirus]‐), KIRC, KIRP, LUAD, LUSC, MESO, and THCA (Figure 5c), and the scatterplot data of ACC, BRCA‐LumA, HNSC, HNSC (HPV‐), KIRC, KIRP, ESCA, and LUAD are shown in Figure 5d. Interestingly, there was no negative correlation between Tregs infiltration and KISS1 expression in pancancers according to the CIBERSORT and CIBERSORT‐ABS algorithms.

Indeed, the infiltration of immunosuppressive cells could be a major reason for undermining the therapeutic efficacy of ICIs (immune checkpoint inhibitors) [26]. Therefore, we further analyzed the correlation of KISS1 expression with eight immune checkpoint pathway genes in diverse cancer types using TCGA [27]. As illustrated in Figure 5e, KISS1 was mostly negatively correlated with the immune checkpoint genes in TGCT, UCEC, STAD, COAD, BLCA, and BRCA and positively correlated with THCA and PRAD. Besides, CD274 had a relatively strong correlation with KISS1 and was mainly negatively related in pancancer, indicating that CD274 may be a potential checkpoint in cancers that have abnormal KISS1 expression [28].

### 3.7. Role of KISS1 in Phenotypic Behavior of Breast Cancer Cells

Based on these results, we conclude a preliminary conclusion that KISS1 affects breast cancers differently from other cancers, which is consistent with previous studies [11]. To further investigate the biological functions and mechanisms of KISS1, we decide to carry out basic experiments using breast cancer cells. First, we explored KISS1 gene expression statuses in different breast cancer lines using the database from CCLE (Cancer Cell Line Encyclopedia) [29] and found that nearly two‐thirds of the cell lines had a high KISS1 gene expression (Figure 6a). We then verified the protein expression status in MDA‐MB‐231 cells, which have a high capacity to metastasize. Indeed, the KISS1 protein expression level was higher in MDA‐MB‐231 cells than in 293T (Figure 6b,c). Importantly, the highly BM derivatives of MDA‐MB‐231 had even higher protein expression levels (Figure 6b,c). Furthermore, we generated KISS1‐knockout (KISS1‐KO) derivatives of MDA‐MB‐231 and MDA‐MB‐231 (BM) cells (Figures 6d, 6e, 6f, and 6g). First, we compared the self‐renewal potential of these cells by assaying their colony formation capacity [6]. KISS1 deficiency reduced the number of cell clones, and ectopic expression of KISS1 in the KISS1‐KO MDA‐MB‐231 cells restored the clone numbers (Figure 6h). We got a similar result in BM derivatives of MDA‐MB‐231 (Figure 6h). These results suggested that KISS1 may promote the stemness of breast cancer cells to some extent. As the tumor‐initiating capacity contributes to the metastasis of a primary tumor, the clone assay may suggest a role of KISS1 in cancer metastasis [5]. We then designed transwell assays to confirm this assumption. From Figure 6i, we can see that the migration ability was significantly reduced when KISS1 was knocked out, whereas ectopic expression of KISS1 restored the migration ability statistically. Together, these results confirm the critical role of KISS1 in promoting the metastasis of breast cancer cells.

Figure 6Role of KISS1 in phenotypic behavior of breast cancer cells. (a) KISS1 expression in different breast cancer cell lines. (b–g) The protein level of KISS1 in breast cancer cells and validation of KISS1 knockout. (h) Colony formation assay reveals the effect of KISS1 on self‐renewal potential of breast cancer cells. (i) Transwell assay detects metastasis ability. (j) WWP1 could bind to KISS1 within breast cancer cells. All panels are the representative results of three independent experiments.

**Figure:**
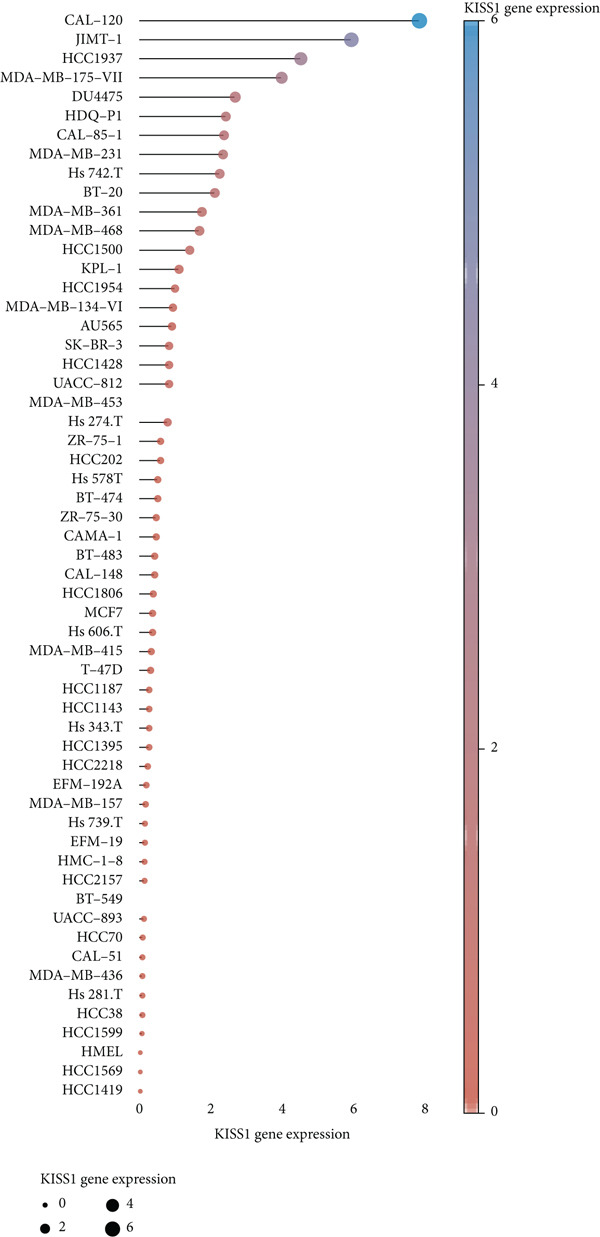
(a)

**Figure:**
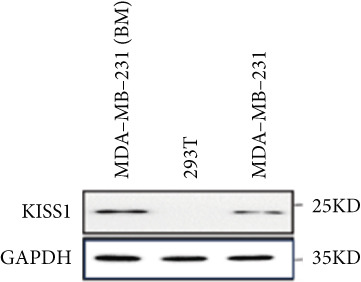
(b)

**Figure:**
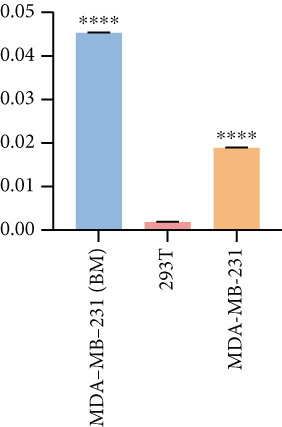
(c)

**Figure:**
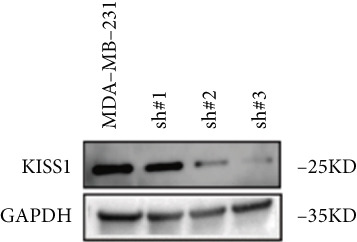
(d)

**Figure:**
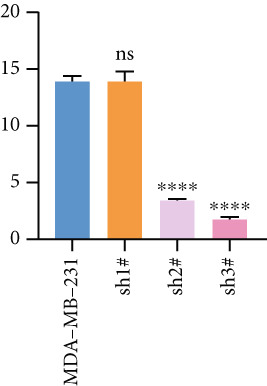
(e)

**Figure:**
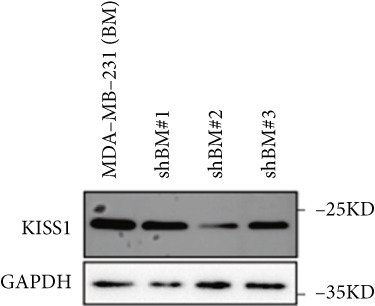
(f)

**Figure:**
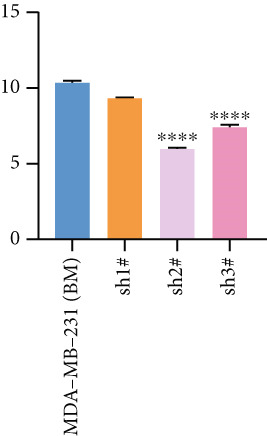
(g)

**Figure:**
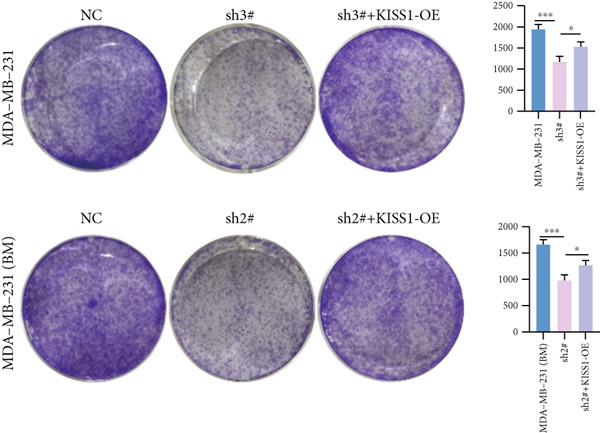
(h)

**Figure:**
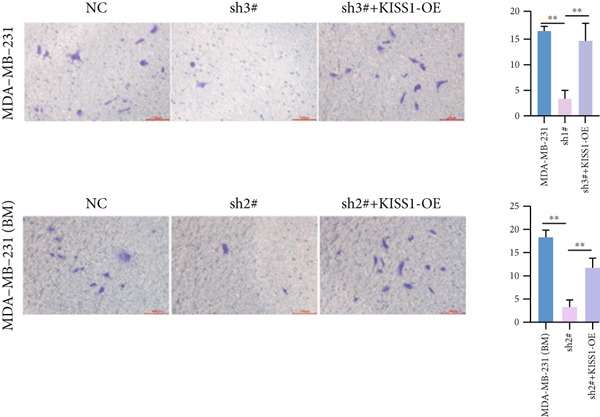
(i)

**Figure:**
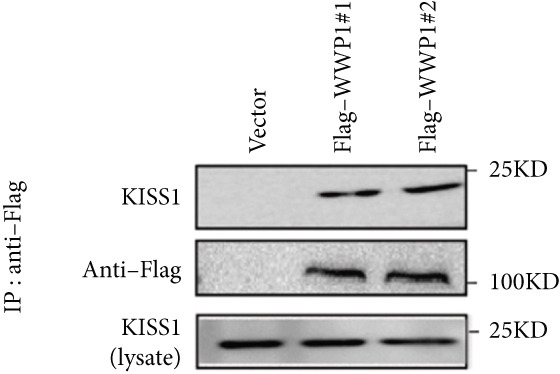
(j)

### 3.8. The Potential PTMs of KISS1 in Tumors

Based on the above results, we were motivated to clarify why the mRNA expression level is different from that of the protein in the online databases. PTMs may play a nonnegligible role in the stability of KISS1 within cells [30]. Indeed, ubiquitination plays a dominant role in the stabilization of proteins [30]. Thus, KISS1 may undergo ubiquitination in a context‐dependent manner, which may explain the difference in its effect on metastasis in different cancers. To test this hypothesis, we performed a binding assay to determine whether some well‐known E3 ubiquitin ligases could bind to KISS1 [31]. Initially, we selected WWP1 (WW domain‐containing E3 ubiquitin protein ligase 1) as the candidate E3 ubiquitin ligase for its well‐known roles in regulating protein ubiquitination [23]. Interestingly, WWP1 could interact with KISS1 in MDA‐MB‐231 cells (Figure 6j). Although a binding assay could not confirm that ubiquitination of KISS1 within cells contributes to the influence of protein stability [31], this indeed indicates the promising role of ubiquitination of KISS1 in cancer therapy.

### 3.9. Knockdown of KISS1 Suppresses Breast Cancer Cell Metastasis In Vivo

To further verify the biological role of KISS1 in the phenotypic behavior of breast cancer cells, we compared KISS1 loss‐ and gain‐of‐function cells for tumor formation in vivo by employing subcutaneous injections. WT or KISS1‐knockdown showed the same tumor volume, which indicated that KISS1 did not affect tumor growth (Figure 7a). We then validated the functional role of KISS1 in bone metastasis using an IIA injection model [32]. KISS1 knockdown significantly reduced cancer cell signal at 2 weeks in the hind limbs of nude mice (Figure 7b). Consistently, KISS1 overexpression in KISS1‐silenced MDA‐MB‐231 cells increased bone metastasis in the same model. These results show that KISS1 promotes breast cancer metastasis by affecting tumor growth in vivo.

Figure 7The biological function of KISS1 in vivo. (a) Left panel: image of tumors in nude mice after injection of KISS1‐knockdown MDA‐MB‐231 cells and control (*n* = 4); right panel: tumor volumes. (b) Left panel: BLI analyses of bone metastasis after IIA injection of different MDA‐MB‐231 cells (*n* = 4 per group; WT = MDA‐MB‐231 cells, BM = bone‐metastatic derivatives, OE = KISS1 overexpression); right panel: BLI quantification of the hind limbs.  ^∗∗∗^ *p* < 0.01 (two‐tailed Student’s *t*‐test).

**Figure:**
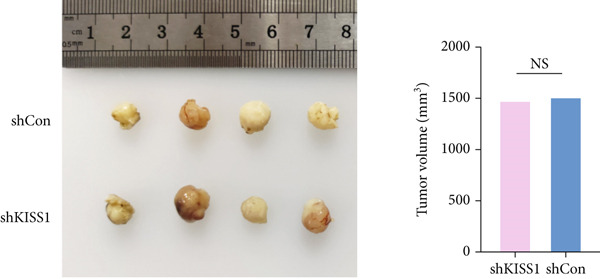
(a)

**Figure:**
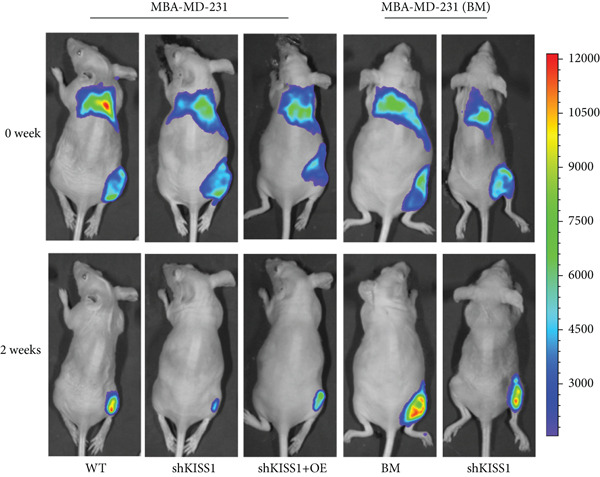
(b)

## 4. Discussion

Metastasis events account for the majority of cancer‐related deaths and challenge the outcomes of tumor therapy [2]. Although immunotherapies have reduced recurrences of various cancers, after prolonged latency, a proportion of patients developed metastases [33]. Given the low response rate of immunotherapy, more potential immunological biomarkers that can regulate tumor metastasis are needed. KISS1 has been reported to be a tumor metastasis suppressor in several cancer types [9, 11, 13]. However, some studies have shown that KISS1 may contribute to breast cancer metastasis [13]. In this study, we analyzed the physiological and pathological effects of KISS1 in pancancer based on TCGA and GTEx databases. First, we confirmed that KISS1 was highly expressed in most cancers, except for KIRP, KIPAN, and LUSC. However, the protein expression levels were not consistent with gene expression status. It could only be detected in breast cancer tissues according to the HPA datasets. These results indicate that KISS1 is highly expressed in breast cancer tissues, but not in other cancer tissues, such as glioma, lung cancer, and pancreatic cancer. This may partly explain the controversial role of KISS1 in the regulation of metastasis in various cancers.

The difference in the expression of KISS1 among various cancers may suggest the biomarker potential of KISS1, and we further tested this potential in the background of clinical and metastatic stages. In the same tumor, KISS1 was expressed differently in various pathological stages. In terms of different metastasis statuses, the difference in KISS1 expression only makes sense in COADREAD, KIPAN, HNSC, PAAD, and SKCM. These results, based on the gene expression database from TCGA, were in contrast to some basic researches [11, 13], which is partly explained by the occurrence of modifications of the KISS1 protein in cancer cells after translation [7].

To our knowledge, this is the first study to analyze the prospective role of KISS1 in pancancer metastasis. Based on the relationship between KISS1 expression and immune cell infiltration, we adopted three immune scores: StromalScore, ImmuneScore, and EstimateScore [32]. In summary, KISS1 correlated strongly with all three scores, and the correlation was mostly negative. For example, in BRCA, the immune cell infiltration level was lower, while there was a higher expression level of KISS1 according to the three immune scores. However, this may partly explain the crucial role of KISS1 in various cancers, including breast cancer. Accordingly, the conclusions presented by the three scores may provide a conceptual map of immune and stromal cells in various cancers, which may explain the differential role of KISS1 in the metastasis of malignancies [22]. Based on this, we next targeted the STM, as the preexistence of immunosuppressive cells is a major reason for the failure of immunotherapy for tumors [4]. We analyzed the potential immunomodulatory effects of KISS1 by evaluating its efficacy on MDSCs and Tregs [34]. Comparisons between these results suggested that the immunosuppressive cells infiltrate highly in BRCA, BRCA‐LumA, KIRC, KIRP, and MESO, where there is high KISS1 expression too. Take BRCA, especially the LumA subtype, for example, the infiltrations of MDSC and Tregs are positively correlated with KISS1 expression according to the TIED, CIBERSORT, and CIBERSORT‐ABS algorithms. It is assumed that this may be related to breast cancer metastasis, especially bone preference [3]. Further investigations into these mechanisms might help to explain the higher frequency of bone metastasis in luminal‐like breast cancer subtypes. Thus, novel effective immunotherapies exclusive of cancer metastasis may come out [4]. However, as the infiltration of immunosuppressive cells may compromise the therapeutic efficacy of ICIs, the study was followed by an analysis of the correlation of KISS1 expression and ICI‐related genes [35]. A significant positive correlation was found. Interestingly, CD274 (PD‐L1) showed a relatively strong correlation with KISS1 expression. Concordantly, PD‐L1 plays an important role in immunotherapy of breast cancers [25], reinforcing the potential role of KISS1 in the treatment of breast cancer.

Given the unique role of KISS1 in breast cancer, we further analyzed whether KISS1 may be an independent predictor of tumor metastasis in patients with breast cancer. We then tested the possibility that knocking down KISS1 could statistically reduce the metastatic capacity of MDA‐MB‐231 cells, which implied that the EMT process of tumor cells was influenced, to some extent, by KISS1.

In summary, our first pancancer analysis and experiments of KISS1, once reported as a metastasis suppressor that expressed differently in various tumors, implied a statistical correlation of KISS1 expression with clinical prognosis; immune cell infiltration, including immunosuppressive cells and lymphocytes; and tumor metastasis capacity, which adds to the understanding of the epigenetic regulatory mechanism in terms of clinical tumor samples. Our study confirmed the therapeutic and prognostic potential of KISS1 and indicates the potential as an immunotherapy target for tumor metastasis. However, the lack of in‐depth clinical and basic experiments restricts the verification of its role as an immunotherapeutic target and calls for further in‐depth studies.

## 5. Conclusions

In this article, we analyzed the potential role of KISS1 in pancancer metastasis using online databases from TCGA, GTEx, and GEO and verified the prometastasic role in breast cancer cells, which is unique to other cancer cells. Besides, we assessed the correlation between KISS1 expression and immune cell infiltration, immunosuppressive cells, and immune checkpoint genes, which may aid in immunotherapy for tumor metastasis. Furthermore, we initially analyzed the potential PTMs of KISS1 within cells, which may affect the phenotype of tumor cells in a text‐dependent manner.

## Consent

The authors have nothing to report.

## Disclosure

All authors approved the submitted manuscript. A preprint has been previously published [36].

## Conflicts of Interest

The authors declare no conflicts of interest.

## Author Contributions

C.W., H.J., and W.X. designed this manuscript and performed the experiments. B.L. and H.Z. drafted this manuscript and prepared the figures. L.Z., Z.L., and J.X. edited and revised the manuscript and provided the project funding. C.W., H.J., and W.X. have contributed equally to this work and share first authorship.

## Funding

This study was funded by the National Natural Science Foundation of China (No. 82372853) to Long Zhang and the Shanghai Magnolia Pujiang Talent Program (No. 23PJ1409800) to Long Zhang.

## Supporting Information

Additional supporting information can be found online in the Supporting Information section.

## Supporting information


**Supporting Information 1** File S1: Abbreviations (word). Alphabetical list of all abbreviations used in the manuscript, with full terms and units.

**Supporting Information 1** File S2: Western blot source data (word). Corresponding original, uncropped western blot images for manuscript blots, with each blot labeled by its corresponding number.

## Data Availability

The original data comes from TCGA (https://portal.gdc.cancer.gov/), GEO (https://www.ncbi.nlm.nih.gov/geo/query/acc.cgi?acc=GSE22138), GTEx (https://commonfund.nih.gov/GTEx/), UCSC (https://xenabrowser.net/), CPTAC (https://proteomic.datacommons.cancer.gov/pdc/), UALCAN (http://ualcan.path.uab.edu/analysis-prot.html), HPA (https://www.proteinatlas.org/), GEPIA2 (http://gepia2.cancer-pku.cn/#analysis), cBioPortal (https://www.cbioportal.org/), GDC (https://portal.gdc.cancer.gov/), and TIMER2 (http://timer.cistrome.org/); public databases and the data were accurate. Other original contributions shown in the study are included in the article and supporting information, and the corresponding authors can be contacted for further inquiries. The reagents, tools, and materials generated in this study are available from the corresponding authors upon request.

## References

[1] Zlotnik A. , Burkhardt A. M. , and Homey B. , Homeostatic Chemokine Receptors and Organ-Specific Metastasis, Nature Reviews Immunology. (2011) 11, no. 9, 597–606, 10.1038/nri3049, 2-s2.0-80052094283. 21866172

[2] Piccolo D. , Nuala V. S. , Shirure Y. B. , Peter Goedegebuure S. , Gholami S. , Hughes C. C. W. , Fields R. C. , and George S. C. , Tumor-on-Chip Modeling of Organ-Specific Cancer and Metastasis, Advanced Drug Delivery Reviews. (2021) 175, 113798, 10.1016/j.addr.2021.05.008. 34015419

[3] Weilbaecher K. N. , Guise T. A. , and McCauley L. K. , Cancer to Bone: A Fatal Attraction, Nature Reviews Cancer. (2011) 11, no. 6, 411–425, 10.1038/nrc3055, 2-s2.0-79957484310. 21593787 PMC3666847

[4] Erin N. , Grahovac J. , Brozovic A. , and Efferth T. , Tumor Microenvironment and Epithelial Mesenchymal Transition as Targets to Overcome Tumor Multidrug Resistance, Drug Resistance Updates. (2020) 53, 100715, 10.1016/j.drup.2020.100715. 32679188

[5] Piskareva O. , Harvey H. , Nolan J. , Conlon R. , Alcock L. , Buckley P. , Dowling P. , O′Sullivan F. , Bray I. , and Stallings R. L. , The Development of Cisplatin Resistance in Neuroblastoma Is Accompanied by Epithelial to Mesenchymal Transition In Vitro, Cancer Letters. (2015) 364, no. 2, 142–155, 10.1016/j.canlet.2015.05.004, 2-s2.0-84930083699. 25960282

[6] Fares J. , Fares M. Y. , Khachfe H. H. , Salhab H. A. , and Fares Y. , Molecular Principles of Metastasis: A Hallmark of Cancer Revisited, Signal Transduction and Targeted Therapy. (2020) 5, no. 1, 10.1038/s41392-020-0134-x. PMC7067809 32296047

[7] Czuba L. C. , Hillgren K. M. , and Swaan P. W. , Post-Translational Modifications of Transporters, Pharmacology & Therapeutics. (2018) 192, 88–99, 10.1016/j.pharmthera.2018.06.013, 2-s2.0-85050385279. 29966598 PMC6263853

[8] Chen L. , Liu S. , and Tao Y. , Regulating Tumor Suppressor Genes: Post-Translational Modifications, Signal Transduction and Targeted Therapy. (2020) 5, no. 1, 10.1038/s41392-020-0196-9. PMC7293209 32532965

[9] Li Z. , Liu J. , Inuzuka H. , and Wei W. , Functional Analysis of the Emerging Roles for the KISS1/KISS1R Signaling Pathway in Cancer Metastasis, Journal of Genetics and Genomics. (2022) 49, no. 3, 181–184, 10.1016/j.jgg.2021.10.005. 34767970

[10] Roa J. , Aguilar E. , Dieguez C. , Pinilla L. , and Tena-Sempere M. , New Frontiers in Kisspeptin/GPR54 Physiology as Fundamental Gatekeepers of Reproductive Function, Frontiers in Neuroendocrinology. (2008) 29, no. 1, 48–69, 10.1016/j.yfrne.2007.07.002, 2-s2.0-37649008207. 17870152

[11] Lee J.-H. , Miele M. E. , Hicks D. J. , Phillips K. K. , Trent J. M. , Weissman B. E. , and Welch D. R. , KiSS-1, a Nhuman Malignant Melanoma Metastasis-Suppressor Gene, JNCI: Journal of the National Cancer Institute. (1996) 88, no. 23, 1731–1737, 10.1093/jnci/88.23.1731, 2-s2.0-0029808561. 8944003

[12] Nash K. T. , Phadke P. A. , Navenot J.-M. , Hurst D. R. , Accavitti-Loper M. A. , Sztul E. , Vaidya K. S. , Frost A. R. , Kappes J. C. , Peiper S. C. , and Eelchl D. R. , Requirement of KISS1 Secretion for Multiple Organ Metastasis Suppression and Maintenance of Tumor Dormancy, Journal of the National Cancer Institute. (2007) 99, no. 4, 309–321, 10.1093/jnci/djk053, 2-s2.0-33847784034. 17312308 PMC1820615

[13] Cvetković D. , Dragan M. , Leith S. J. , Mir Z. M. , Leong H. S. , Pampillo M. , Lewis J. D. , Babwah A. V. , and Bhattacharya M. , KISS1R Induces Invasiveness of Estrogen Receptor-Negative Human Mammary Epithelial and Breast Cancer Cells, Endocrinology. (2013) 154, no. 6, 1999–2014, 10.1210/en.2012-2164, 2-s2.0-84878428917. 23525242

[14] Saidak Z. , Soudet S. , Lottin M. , Salle V. , Sevestre M.-A. , Clatot F. , and Galmiche A. , A Pan-Cancer Analysis of the Human Tumor Coagulome and Its Link to the Tumor Immune Microenvironment, Cancer Immunology, Immunotherapy. (2021) 70, no. 4, 923–933, 10.1007/s00262-020-02739-w. 33057845 PMC10991611

[15] Zhang K. and Hong W. A. N. G. , Cancer Genome Atlas Pan-Cancer Analysis Project, Zhongguo Fei Ai Za Zhi. (2015) 18, no. 4. 10.3779/j.issn.1009-3419.2015.04.02 PMC6000284 25936886

[16] Chen F. , Chandrashekar D. S. , Varambally S. , and Creighton C. J. , Pan-Cancer Molecular Subtypes Revealed by Mass-Spectrometry-Based Proteomic Characterization of More Than 500 Human Cancers, Nature Communications. (2019) 10, no. 1, 10.1038/s41467-019-13528-0. PMC6908580 31831737

[17] Song X. , Rao D. , Gui H. , Zhou M. , Zhong W. , Mao C. , and Ma J. , Identification of Potential Hub Genes Related to the Progression and Prognosis of Hepatocellular Carcinoma Through Integrated Bioinformatics Analysis, Oncology Reports. (2020) 43, no. 1, 133–146. 31746405 10.3892/or.2019.7400 PMC6908929

[18] Tang Z. , Kang B. , Li C. , Chen T. , and Zhang Z. , GEPIA2: An Enhanced Web Server for Large-Scale Expression Profiling and Interactive Analysis, Nucleic Acids Research. (2019) 47, no. W1, W556–W560, 10.1093/nar/gkz430, 2-s2.0-85069235274. 31114875 PMC6602440

[19] Gao J. , Aksoy B. A. , Dogrusoz U. , Dresdner G. , Benjamin Gross S. , Sumer O. , Sun Y. , Jacobsen A. , Sinha R. , Larsson E. , Cerami E. , Sander C. , and Schultz N. , Integrative Analysis of Complex Cancer Genomics and Clinical Profiles Using the cBioPortal, Science Signaling. (2013) 6, no. 269, pl1–pl1, 10.1126/scisignal.2004088, 2-s2.0-84875740314. 23550210 PMC4160307

[20] Li T. , Fu J. , Zeng Z. , Cohen D. , Li J. , Chen Q. , Li B. , and Liu X. S. , TIMER2. 0 for Analysis of Tumor-Infiltrating Immune Cells, Nucleic Acids Research. (2020) 48, no. W1, W509–W514, 10.1093/nar/gkaa407. 32442275 PMC7319575

[21] Yoshihara K. , Shahmoradgoli M. , Martínez E. , Vegesna R. , Kim H. , Torres-Garcia W. , Treviño V. , Shen H. , Laird P. W. , Levine D. A. , Carter S. L. , Getz G. , Stemke-Hale K. , Mills G. B. , and Verhaak R. G. W. , Inferring Tumour Purity and Stromal and Immune Cell Admixture From Expression Data, Nature Communications. (2013) 4, no. 1, 10.1038/ncomms3612, 2-s2.0-84885673911. PMC3826632 24113773

[22] Thorsson V. , Gibbs D. L. , Brown S. D. , Wolf D. , Bortone D. S. , Yang T.-H. O. , Porta-Pardo E. , Gao G. F. , Plaisier C. L. , Eddy J. A. , and Ziv E. , The Immune Landscape of Cancer, Immunity. (2018) 48, no. 4, 812–830. 29628290 10.1016/j.immuni.2018.03.023 PMC5982584

[23] Lee Y.-R. , Chen M. , Lee J. D. , Zhang J. , Lin S.-Y. , Tian-Min F. , Chen H. , Ishikawa T. , Chiang S.-Y. , Katin J. , Zhang Y. , Shulga Y. V. , Bester A. C. , Fung J. , Monteleone E. , Wan L. , Shen C. , Hsu C.-H. , Paga A. , Clohessy J. G. , Teruya-Feldstein J. , Jain S. , Hao W. , Matesic L. , Chen R.-H. , Wen W. , and Pandolfi P. P. , Reactivation of PTEN Tumor Suppressor for Cancer Treatment Through Inhibition of a MYC-WWP1 Inhibitory Pathway, Science. (2019) 364, no. 6441, eaau0159, 10.1126/science.aau0159, 2-s2.0-85066443035. 31097636 PMC7081834

[24] Wei C. , Wang B. , Peng D. , Zhang X. , Li Z. , Luo L. , Yingjie H. L. , Xuezhi D. , Li S. , Zhang S. , Zhang Z. , Han L. , and Zhang J. , Pan-Cancer Analysis Shows That ALKBH5 Is a Potential Prognostic and Immunotherapeutic Biomarker for Multiple Cancer Types Including Gliomas, Frontiers in Immunology. (2022) 13, 849592, 10.3389/fimmu.2022.849592. 35444654 PMC9013910

[25] Kim J. M. and Chen D. S. , Immune Escape to PD-L1/PD-1 Blockade: Seven Steps to Success (or Failure), Annals of Oncology. (2016) 27, no. 8, 1492–1504, 10.1093/annonc/mdw217, 2-s2.0-84984973625. 27207108

[26] Yan Y. , Chen B. , Yin Q. , Wang Z. , Yang Y. , Wan F. , Wang Y. , Tang M. , Xia H. , Chen M. , Liu J. , Wang S. , Zhang Q. , and Wang Y. , Dissecting Extracellular and Intracellular Distribution of Nanoparticles and Their Contribution to Therapeutic Response by Monochromatic Ratiometric Imaging, Nature Communications. (2022) 13, no. 1, 10.1038/s41467-022-29679-6. PMC9010411 35422063

[27] Kalbasi A. and Ribas A. , Tumour-Intrinsic Resistance to Immune Checkpoint Blockade, Nature Reviews Immunology. (2020) 20, no. 1, 25–39, 10.1038/s41577-019-0218-4. PMC8499690 31570880

[28] Huang X. , Qi Zhang Y. , Lou J. W. , Zhao X. , Wang L. , Zhang X. , Li S. , Zhao Y. , Chen Q. , Liang T. , and Bai X. , USP22 Deubiquitinates CD274 to Suppress Anticancer Immunity, Cancer Immunology Research. (2019) 7, no. 10, 1580–1590. 31399419 10.1158/2326-6066.CIR-18-0910

[29] Nusinow D. P. , Szpyt J. , Ghandi M. , Rose C. M. , Robert McDonald E. , Kalocsay M. , Jané-Valbuena J. , Gelfand E. , Devin K. , Schweppe M. J. , Golji J. , Porter D. A. , Tomas Rejtar Y. , Wang K. , Kryukov G. V. , Stegmeier F. , Erickson B. k. , Garraway L. A. , Sellers W. R. , and Gygi S. P. , Quantitative Proteomics of the Cancer Cell Line Encyclopedia, Cell. (2020) 180, no. 2, 387–402. 31978347 10.1016/j.cell.2019.12.023 PMC7339254

[30] Wang S. , Osgood A. O. , and Chatterjee A. , Uncovering Post-Translational Modification-Associated Protein–Protein Interactions, Current Opinion in Structural Biology. (2022) 74, 102352, 10.1016/j.sbi.2022.102352. 35334254 PMC9464464

[31] Buetow L. and Huang D. T. , Structural Insights Into the Catalysis and Regulation of E3 Ubiquitin Ligases, Nature Reviews Molecular Cell Biology. (2016) 17, no. 10, 626–642, 10.1038/nrm.2016.91, 2-s2.0-84980329401. 27485899 PMC6211636

[32] Wang Q. , Li Z. , Zhou S. , Li Z. , Huang X. , He Y. , Zhang Y. , Zhao X. , Tang Y. , and Min X. , NCAPG2 Could Be an Immunological and Prognostic Biomarker: From Pan-Cancer Analysis to Pancreatic Cancer Validation, Frontiers in Immunology. (2023) 14, 1097403, 10.3389/fimmu.2023.1097403. 36776838 PMC9911455

[33] Hutzen B. , Paudel S. N. , Kararoudi M. N. , Cassady K. A. , Lee D. A. , and Cripe T. P. , Immunotherapies for Pediatric Cancer: Current Landscape and Future Perspectives, Cancer and Metastasis Reviews. (2019) 38, no. 4, 573–594, 10.1007/s10555-019-09819-z. 31828566 PMC6994452

[34] Sakaguchi S. , Mikami N. , Wing J. B. , Tanaka A. , Ichiyama K. , and Ohkura N. , Regulatory T Cells and Human Disease, Annual Review of Immunology. (2020) 38, no. 1, 541–566. 10.1146/annurev-immunol-042718-041717 32017635

[35] Bagchi S. , Yuan R. , and Engleman E. G. , Immune Checkpoint Inhibitors for the Treatment of Cancer: Clinical Impact and Mechanisms of Response and Resistance, Annual Review of Pathology: Mechanisms of Disease. (2021) 16, no. 1, 223–249, 10.1146/annurev-pathol-042020-042741. 33197221

[36] Jiang H. , Xiao J. , Zhang H. , Li B. , Xu W. , and Li Z. , KISS1 Is a Potential Immunological and Prognostic Biomarker for Cancer Metastasis in Pan-Cancer and Affects Breast Cancer in a Different Way, [Preprint].bioRxiv,2023-07-07,URL: https://www.biorxiv.org/node/3242961.

