# Supplementary material for: Pancancer Analyses of KISS1 as a Potential Biomarker for Tumor Metastasis and Immunotherapy and Therapeutic Target for Breast Cancer
Source: Int J Genomics. 2026 Jan 8;2026:5902518. doi: 10.1155/ijog/5902518 (PMC12780545; doi:10.1155/ijog/5902518)
Supplement: Supplementary file 1 — Supporting Information 1 File S1: Abbreviations (word). Alphabetical list of all abbreviations used in the manuscript, with full terms and units. [file IJOG-2026-5902518-s002.docx]

**Abbreviations**

ACC Adrenocortical carcinoma

BLCA Bladder Urothelial Carcinoma

BRCA Breast invasive carcinoma

CESC Cervical squamous cell carcinoma and endocervical adenocarcinoma

CHOL Cholangiocarcinoma

COAD Colon adenocarcinoma

COADREAD Colon adenocarcinoma/Rectum adenocarcinoma Esophageal carcinoma

DLBC Lymphoid Neoplasm Diffuse Large B-cell Lymphoma

ESCA Esophageal carcinoma

FPPP FFPE Pilot Phase II

GBM Glioblastoma multiforme

GBMLGG Glioma

HNSC Head and Neck squamous cell carcinoma

KICH Kidney Chromophobe

KIPAN Pan-kidney cohort (KICH+KIRC+KIRP)

KIRC Kidney renal clear cell carcinoma

KIRP Kidney renal papillary cell carcinoma

LAML Acute Myeloid Leukemia

LGG Brain Lower Grade Glioma

LIHC Liver hepatocellular carcinoma

LUAD Lung adenocarcinoma

LUSC Lung squamous cell carcinoma

MESO Mesothelioma

OV Ovarian serous cystadenocarcinoma

PAAD Pancreatic adenocarcinoma

PCPG Pheochromocytoma and Paraganglioma

PRAD Prostate adenocarcinoma

READ Rectum adenocarcinoma

SARC Sarcoma

STAD Stomach adenocarcinoma

SKCM Skin Cutaneous Melanoma

STES Stomach and Esophageal carcinoma

TGCT Testicular Germ Cell Tumors

THCA Thyroid carcinoma

THYM Thymoma

UCEC Uterine Corpus Endometrial Carcinoma

UCS Uterine Carcinosarcoma

UVM Uveal Melanoma

OS Osteosarcoma

ALL Acute Lymphoblastic Leukemia

NB Neuroblastoma

WT High-Risk Wilms Tumor

TCGA The Cancer Genome Atlas

GTEx Genotype-Tissue Expression
