# Supplementary figures and images for: Pancancer Analyses of KISS1 as a Potential Biomarker for Tumor Metastasis and Immunotherapy and Therapeutic Target for Breast Cancer
Source: Int J Genomics. 2026 Jan 8;2026:5902518. doi: 10.1155/ijog/5902518 (PMC12780545; doi:10.1155/ijog/5902518)

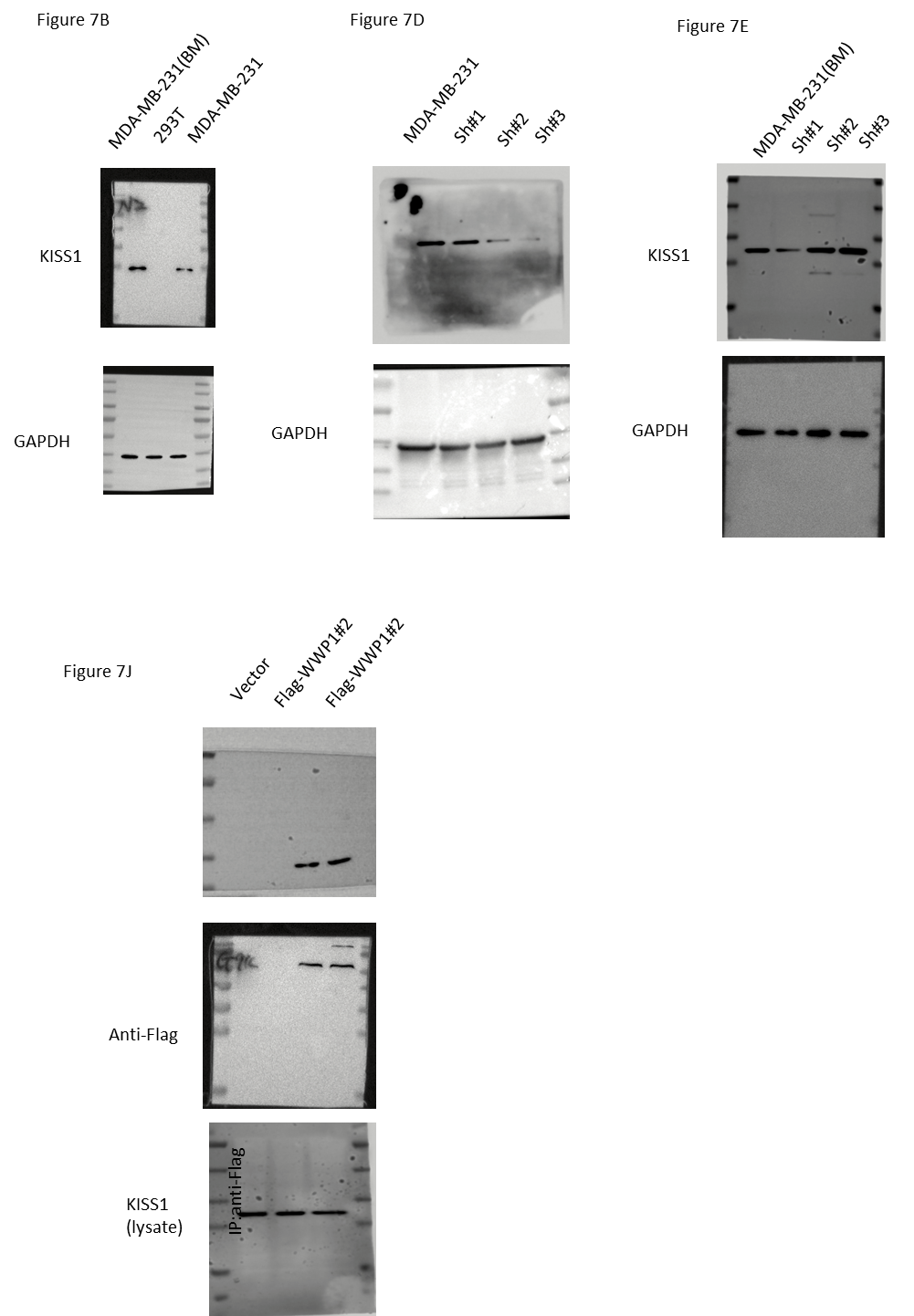

Supplement: Supplementary file 2 — Supporting Information 1 File S2: Western blot source data (word). Corresponding original, uncropped western blot images for manuscript blots, with each blot labeled by its corresponding number. [file IJOG-2026-5902518-s001.docx]
